# Proteome-defined changes in cellular pathways for decidua and trophoblast tissues associated with location and viability of early-stage pregnancy

**DOI:** 10.1186/s12958-022-00908-3

**Published:** 2022-02-21

**Authors:** Lynn A. Beer, Suneeta Senapati, Mary D. Sammel, Kurt T. Barnhart, Courtney A. Schreiber, David W. Speicher

**Affiliations:** 1 Center for Systems & Computational Biology, The Wistar Institute, Philadelphia, PA USA; 2 Department of Obstetrics and Gynecology, University of Pennsylvania, Philadelphia, PA USA; 3 Department of Biostatistics and Informatics, Colorado School of Public Health, Aurora, CO USA; 4 Department of Biostatistics, Epidemiology and Informatics, University of Pennsylvania, Philadelphia, PA USA

**Keywords:** Ectopic pregnancy, Early pregnancy loss, Decidua, Trophoblast, Maternal–fetal interface, Proteomics

## Abstract

**Background:**

In early pregnancy, differentiating between a normal intrauterine pregnancy (IUP) and abnormal gestations including early pregnancy loss (EPL) or ectopic pregnancy (EP) is a major clinical challenge when ultrasound is not yet diagnostic. Clinical treatments for these outcomes are drastically different making early, accurate diagnosis imperative. Hence, a greater understanding of the biological mechanisms involved in these early pregnancy complications could lead to new molecular diagnostics.

**Methods:**

Trophoblast and endometrial tissue was collected from consenting women having an IUP (n = 4), EPL (*n* = 4), or EP (*n* = 2). Samples were analyzed by LC–MS/MS followed by a label-free proteomics analysis in an exploratory study. For each tissue type, pairwise comparisons of different pregnancy outcomes (EPL vs. IUP and EP vs. IUP) were performed, and protein changes having a fold change ≥ 3 and a Student’s t-test p-value ≤ 0.05 were defined as significant. Pathway and network classification tools were used to group significantly changing proteins based on their functional similarities.

**Results:**

A total of 4792 and 4757 proteins were identified in decidua and trophoblast proteomes. For decidua, 125 protein levels (2.6% of the proteome) were significantly different between EP and IUP, whereas EPL and IUP decidua were more similar with only 68 (1.4%) differences. For trophoblasts, there were 66 (1.4%) differences between EPL and IUP. However, the largest group of 344 differences (7.2%) was observed between EP and IUP trophoblasts. In both tissues, proteins associated with ECM remodeling, cell adhesion and metabolic pathways showed decreases in EP specimens compared with IUP and EPL. In trophoblasts, EP showed elevation of inflammatory and immune response pathways.

**Conclusions:**

Overall, differences between an EP and IUP are greater than the changes observed when comparing ongoing IUP and nonviable intrauterine pregnancies (EPL) in both decidua and trophoblast proteomes. Furthermore, differences between EP and IUP were much higher in the trophoblast than in the decidua. This observation is true for the total number of protein changes as well as the extent of changes in upstream regulators and related pathways. This suggests that biomarkers and mechanisms of trophoblast function may be the best predictors of early pregnancy location and viability.

**Supplementary Information:**

The online version contains supplementary material available at 10.1186/s12958-022-00908-3.

## Background

During early pregnancy, differentiating between an ongoing healthy intrauterine pregnancy (IUP) and abnormal conditions when ultrasound is not yet diagnostic is a major clinical challenge. Women who present with abdominal pain and/or vaginal bleeding will ultimately be diagnosed with: 1) an ongoing IUP, 2) a miscarriage or early pregnancy loss (EPL), or 3) an ectopic pregnancy (EP). EPL affects 10%–20% of pregnancies [1], while EP occurs in 1–2% of pregnant women and is a leading cause of maternal mortality and morbidity accounting for 6% of pregnancy-related deaths [2, 3]. Appropriate clinical treatment for these three outcomes is drastically different making early, accurate diagnosis imperative. Currently, progesterone and human chorionic gonadotrophin (hCG) are the most commonly used serum biomarkers for evaluating pregnancy prognosis when ultrasound is inconclusive [4, 5]. Similarly, serial hCG levels and pelvic ultrasound are the standard methods of diagnosing an EP. However, neither method can predict an EP with sufficient and reproducible accuracy and importantly, there is currently no diagnostic method that is able to distinguish between both pregnancy location and viability at very early stages [3, 6]. Therefore, a better understanding of the basic mechanisms of normal and abnormal implantation could have profound clinical significance.

The maternal–fetal interface consists of the maternal endometrium and the invading fetal trophoblast. A coordinated balance between the invading trophoblast and a receptive maternal decidua is necessary for maintaining a successful pregnancy [7, 8]. In preparation for pregnancy, the endometrium is transformed into a structure called decidua. This occurs under the regulation of the ovarian hormones estrogen and progesterone in a complex morphological and biochemical alteration process called decidualization [9, 10]. Additionally, the presence of an embryo in the uterus activates specific molecular and cellular responses within the decidua [11, 12]. Embryo implantation is a highly coordinated process where the trophoblast establishes contact with the decidua and a regulated cascade of growth factors, cytokines, and hormones occurs via embryonic as well as maternal tissues of both uterine and extrauterine origins [12]. Alterations in the implantation process can lead to adverse pregnancy complications including infertility, miscarriage, fetal growth restriction and preeclampsia [13]. However, the functional changes that occur in the decidua and trophoblast in these and other pregnancy complications such EP are not fully understood. In this study, we used unbiased proteomics in an exploratory study to analyze tissue specimens from the maternal–fetal interface from women with a viable IUP (as determined by ultrasound), or a nonviable EPL or EP to identify significant changes associated with early pregnancy and/or location. A greater understanding of the biological mechanisms in these early pregnancy complications could lead to new, practice changing molecular diagnostics.

## Materials and methods

### Patients and tissue specimens

After informed consent was obtained from participants, tissue was collected from gestational age (GA)- matched women having an IUP (*n* = 4), EPL (*n* = 4), or EP (*n* = 2). Specifically, tissue was obtained from the operating room at the time of scheduled clinical procedure including scheduled elective termination of pregnancy, uterine evacuation for pregnancy loss or laparoscopic treatment for EP. In the cases of EP an endometrial sampling with a pippelle catheter was performed in the operating room. Tissues were exampled using a dissection microscope and rinsed with saline solution to allow identification of desired tissue (gestational sac, decidua, fallopian tube mucosa or endometrium). Tissue was divided into 5 – 7 mm aliquots using a sterile scalpel and snap-frozen using liquid nitrogen within 30 min of removal from the patient. Patient characteristics for the study sample are shown in Table 1.

**Table 1:** Patient characteristics of the study sample

Patient	Age (yrs)	Race	Gravidity	# Prior Full- Term Births	# Prior Pre-Term Births	# Prior First Tri-mester Loss	# Prior Pregnancy termination	Final GA (days)	Decidua	Trophoblast
**IUP-A**	36	African American	6	2	0	3	0	66	**A**	**A**
**IUP-B**	22	White	1	0	0	0	0	50	**B**	**B**
**IUP-C**	27	African American	3	1	0	1	1	48	**C**	**C**
**IUP-D**	34	White	1	0	0	0	0	63		**D**
**EPL-A**	24	White	8	3	0	3	1	64	**A**	**A**
**EPL-B**	35	White	5	3	0	1	0	54	**B**	**B**
**EPL-C**	23	Other	3	1	0	1	0	42	**C**	**C**
**EPL-D**	29	African American	4	0	1	2	0	63		**D**
**EP-A**	28	African American	4	2	1	0	0	48	**A**	**A**
**EP-B**	35	White	2	1	0	0	0	NA	**B**	**B**

### Tissue sample preparation

Small sections of frozen tissue biopsies (~ 20–40 mg) were cryo-homogenized using a liquid nitrogen-cooled mini-mortar. Ground tissue was weighed, resuspended in a Tris-SDS lysis buffer (1% SDS, 50 mM Tris–HCL, 150 mM NaCl, 1 mM EDTA, 0.15 mM PMSF, 1 µg/ml leupeptin, 1 µg/ml pepstatin), sonicated on ice for 2 × 30 s using a probe-tip sonicator with a 30 s period of cooling on ice in between to prevent sample warming above 0–4 °C, and centrifuged for 10 min at 12,000 × g at 4 °C. Supernatants were recovered, and protein concentrations were measured using a BCA assay (Pierce). Lysates were then aliquoted, snap-frozen using liquid nitrogen, and stored at -80 °C until use.

### SDS-PAGE/ In-gel Trypsin Digestion

Frozen tissue lysates were thawed briefly, resuspended in SDS-PAGE sample buffer and run for a short distance (0.5 cm) onto pre-cast NUPAGE (Thermo Fisher Scientific) 1-D SDS gels. Gels were stained with Colloidal Blue (Thermo Fisher Scientific) and the entire 0.5 cm stained gel region was excised and digested overnight using 20 ng/ml modified trypsin, as previously described [14].

### Liquid chromatography-tandem mass spectrometry (LC–MS/MS)

Samples were analyzed on a Q Exactive HF mass spectrometer (Thermo Scientific) equipped with a Nano-Acquity ultrahigh pressure liquid chromatography (UPLC) System (Waters, Milford, MA) with the column heater maintained at 40 °C. Tryptic digests were injected onto a UPLC Symmetry trap column (180 µm i.d. × 2 cm packed with 5 µm C18 resin; Waters), and peptides were separated by reverse phase-high pressure liquid chromatography (RP-HPLC) on a BEH C18 nanocapillary analytical column (75 µm i,d, × 25 cm, 1.7 µm particle size, Waters). Solvent A was Milli-Q (Millipore, Billerica, MA) water containing 0.1% formic acid, and Solvent B was acetonitrile containing 0.1% formic acid. Peptides were eluted at 200 nl/min using an acetonitrile gradient consisting of 5–30% B over 225 min, 30–80% B over 5 min, 80% B for 10 min before returning to 5% B over 0.5 min. The column was re-equilibrated using 5% B at 300 nl/min for 5 min before injecting the next sample. To minimize carryover, a blank was run between each experimental sample by injecting water and using a 30 min gradient with the same solvents. The full MS scan was acquired in profile mode at 60,000 resolution with a 400–2000 m/z scan range. Data-dependent MS/MS was performed on the top 20 most abundant precursor ions in every full MS scan. Unassigned and + 1 charge ions were rejected, and peptide match was set to preferred. Precursor ions subjected to MS/MS were excluded from repeated analysis for 20 s.

### Data analysis

Raw mass spectrometric data from the trophoblast and decidua datasets were processed using MaxQuant (Ver. 1.5.2.8) [15] in two separate analyses. The "match between runs" option to match identifications across samples based on accurate m/z and retention times was enabled with 0.7 min match time window and 10 min alignment time window [16], and peak lists were searched against the human Uniprot database (released 06/29/2017; 159,743 entries) with a full tryptic constraint using the Andromeda search engine [17]. Precursor mass tolerance was set to 4.5 ppm in the main search, and fragment mass tolerance was set to 20 ppm. A maximum of two-missed cleavages was allowed, and minimal peptide length was set to seven amino acids. Carbamidomethyl cysteine was set as a fixed modification and methionine oxidation and N-terminus acetylation were set as variable modifications. A database of common expected contaminants including keratins and trypsin, as well as a decoy database produced by reversing the sequence of each protein, were appended to the forward Uniprot database and this combined database was used for searches. Criteria for high confidence peptide/protein identifications utilized false discovery rates (FDR) of 1% for proteins and peptides. Relative abundance of each protein across all samples in an experiment was determined using the label-free quantitation (LFQ) option of MaxQuant [18]. Protein database entries that shared all identified peptides were combined into a single protein group by the MaxQuant software. In cases where all identified peptides from a protein were a subset of identified peptides from another protein, these proteins were also combined into that protein group. Peptides that matched multiple protein groups (i.e.,“razor” peptides) were assigned to the protein group with the most unique peptides. Quantification was performed using unique peptides, including those modified by acetylation (protein N-terminal) and oxidation (Met). A minimum peptide ratio of 1 was required for protein intensity normalization, and “Fast LFQ” was enabled [18].

Protein identifications were filtered using Perseus software (Ver. 1.6.0.2; http://www.perseus-framework.org) [19] to remove decoy database reverse identifications, contaminants, and proteins identified only by site modified peptides or proteins identified by a single uniquely-mapping peptide. In addition, prior to statistical analysis, protein group LFQ intensities were log2 transformed to reduce the impact of outliers. To reduce quantitative uncertainty, protein groups were removed if they had less than three valid intensity values present in at least one of the categorical groups, i.e. either viable IUP or nonviable pregnancy (EP and EPL). Prior to statistical analysis, Perseus was used to impute missing data points by creating a downshifted Gaussian distribution of random numbers to simulate the distribution of low signal values (imputation width = 0.3, shift = 1.8). Perseus was also used for hierarchical clustering and data visualization using volcano plots.

### Statistical analysis

Statistical analyses were performed using Perseus software or Microsoft Excel 2016. Significantly changed proteins were defined as having both ≥ threefold change between the average of all tissue samples for each pregnancy outcome, and a Student t-test p-value ≤ 0.05. Heat maps of protein intensity z-scores were used to visualize proteins which displayed increased or decreased protein levels between pregnancy outcomes.

### Functional pathway and network classification

Several bioinformatic tools were used to group significantly changing proteins based on their functional similarity including: The Database for Annotation, Visualization, and Integrated Discovery (DAVID, Ver.6.8; http://david.ncifcrf.gov) [20, 21]; STRING (Ver. 11; http://string-db.org/) [22]; and Ingenuity Pathway Analysis (IPA, Qiagen Inc.; www.qiagenbioinformatics.com/products/ingenuity-pathway-analysis) [23]. Specifically, DAVID was used for KEGG (Kyoto Encyclopedia of Genes and Genomes) database pathway enrichment analysis www.genome.jp/kegg/pathway.html [24]. For each tissue dataset pairwise comparison (EP vs. IUP and EPL vs. IUP), gene sets of significant proteins having ≥ threefold increases or decreases were input and all proteins identified in the respective decidua or trophoblast datasets were used as the background proteome. Benjamini–Hochberg adjusted p-value ≤ 0.05 was considered significant. In addition, protein–protein interaction networks were obtained from STRING with the highest confidence interaction score setting (> 0.9), and experiments and databases were selected as the active interaction sources. STRING networks were generated for pairwise comparisons of both tissue-types, and significantly increasing or decreasing protein sets were input separately as described above. Gene Ontology (GO) [25, 26] functional enrichment of biological processes was performed within STRING using all proteins identified in the respective decidua or trophoblast datasets as the background proteomes, and FDR ≤ 0.05 was considered significant.

For IPA analyses, separate Core Analyses were performed for the decidua and trophoblast datasets. Uniprot protein identifiers and corresponding fold changes and p-values were uploaded into the application and each identifier was mapped to its corresponding object in Ingenuity's Knowledge Base. A fold-change cutoff of ≥ 3 and p-value ≤ 0.05 were set to identify significant changes, and each respective tissue dataset was set as the reference background. Additionally, the Upstream Regulator Analysis analytic was used to determine likely upstream regulators that are connected to dataset genes through a set of direct or indirect relationships. Upstream regulators were limited to genes, RNAs, proteins, and endogenous mammalian chemicals and regulators having activation z-scores ≥ 2 (activated) or ≤ -2 (inhibited), and right-tailed Fisher’s Exact Test p-value ≤ 0.05 were significant. Also, the IPA Regulator Effects analytic was used to provide further insight into the causes and effects of significantly changing genes or proteins in a dataset by merging Upstream Regulator networks with Downstream Effects networks [23]. Regulator Effects networks were generated using a z-score cutoff of 2 and a p-value cutoff of 0.01. Finally, a Comparison Analysis was performed and comparison heatmaps were generated within IPA to visualize changes across all experimental comparisons simultaneously.

### Cell type distribution in specimens

To determine the degree of similarities among cell types for the different pregnancy tissues, we cross-referenced our tissue proteomes with a single-cell RNA sequencing (scRNA-seq) analysis of human first-trimester placental villi and decidual cells [27]. The published data file containing the “Average Expression Profiles of All Cell Types of Placenta” from Suryawanshi et al. was input into Perseus and the trophoblast and decidua tissue proteome datasets were matched to the corresponding reference datasets by matching gene names. Hierarchical clustering with Euclidian distance and k-means pre-processing was performed to group proteins into clusters of cell types where the reference dataset expression profiles were highest.

## Results

### Proteome analysis of early pregnancy tissues shows substantial consistency between clinical outcomes and between tissue types

**Fig. 1:**
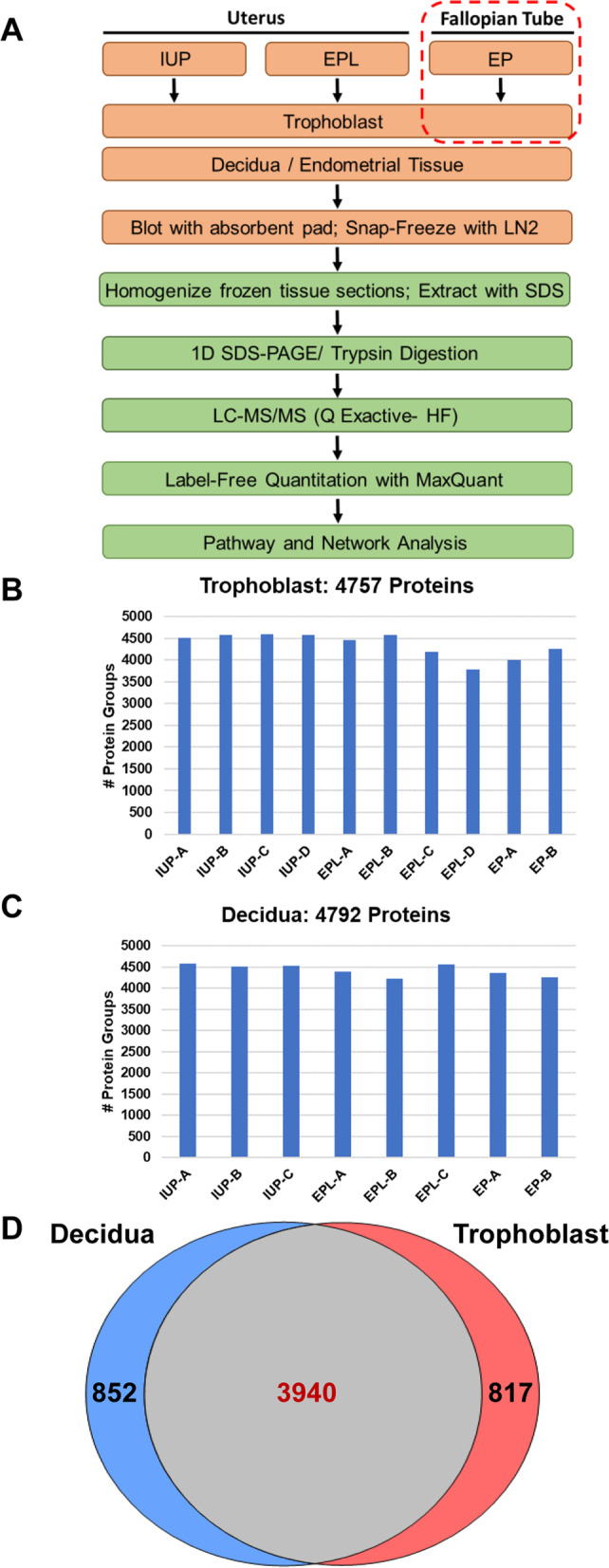
Proteome analysis of placental tissue specimens. **a.** Scheme for processing and analysis of placental tissue specimens. As indicated, all tissue specimens had a uterine location with the exception of the EP trophoblasts. **b.** Numbers of high confidence protein identifications in trophoblast specimens. **c.** Numbers of high confidence protein identifications in decidua specimens. **d.** Overlap of protein identifications in placental tissue proteomes

The flow diagram in Fig. 1a summarizes tissue location, sample processing and analysis of specimens. Two decidua specimens (IUP-D and EPL-D, Table 1) were eliminated due to high blood contamination in the tissue, which greatly reduced depth of analysis and protein identifications for those samples. A total of 4757 and 4792 high confidence proteins, respectively, were identified in the remaining ten trophoblast and eight decidua proteomes (Fig. 1b and c). There was substantial similarity of protein identifications across the two tissue types with > 82% overlap (Fig. 1d). Similarly, protein identification consistency was high across biological specimens for each tissue and pregnancy outcome with > 80% overlap within each group, with the exception of EPL trophoblasts which showed a slightly lower overlap of ~ 73% (Supplementary Figs S1a-c and S2a-c). The combined sample group protein compositions from all three outcomes were extremely similar with > 90% overlap in both tissue types (Supplementary Figs S1d and S2d).

### Proteome differences were greater for comparisons of embryo location than viability of intrauterine pregnancies for both trophoblast and decidua tissues

**Fig. 2:**
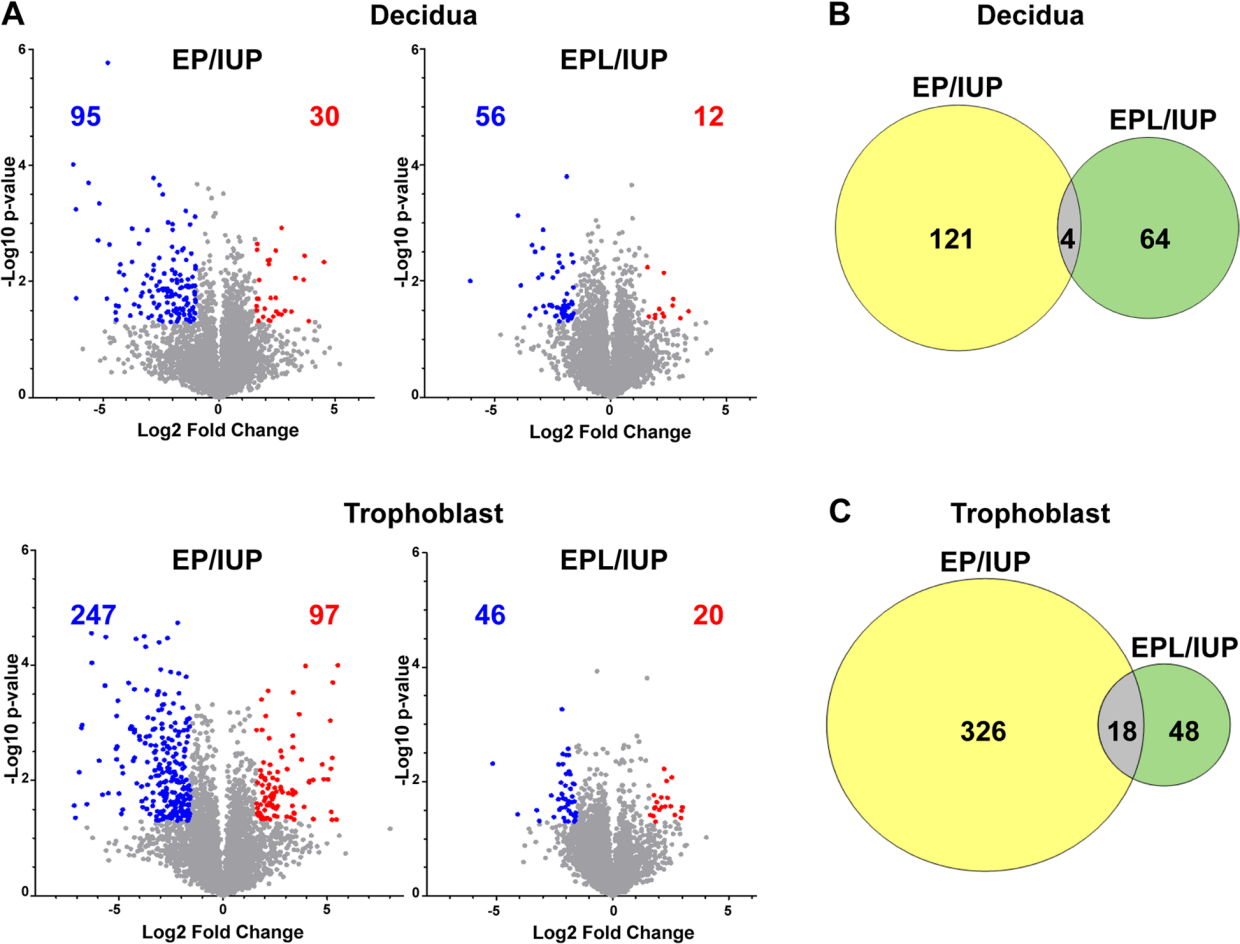
Significant changes in decidua and trophoblast tissue proteomes. **a.** Volcano plots for the indicated pairwise comparisons using –log10 p-values vs. log2 fold change. Significantly changing proteins (Fold change ≥ 3 and Student’s t-test p-value ≤ 0.05) are highlighted. Red =  ≥ threefold increase; Blue =  ≥ threefold decrease. **b.** Overlap of significantly changing proteins for EP/IUP and EPL/IUP decidua. **c.** Overlap of significantly changing proteins for EP/IUP and EPL/IUP trophoblasts

**Fig. 3:**
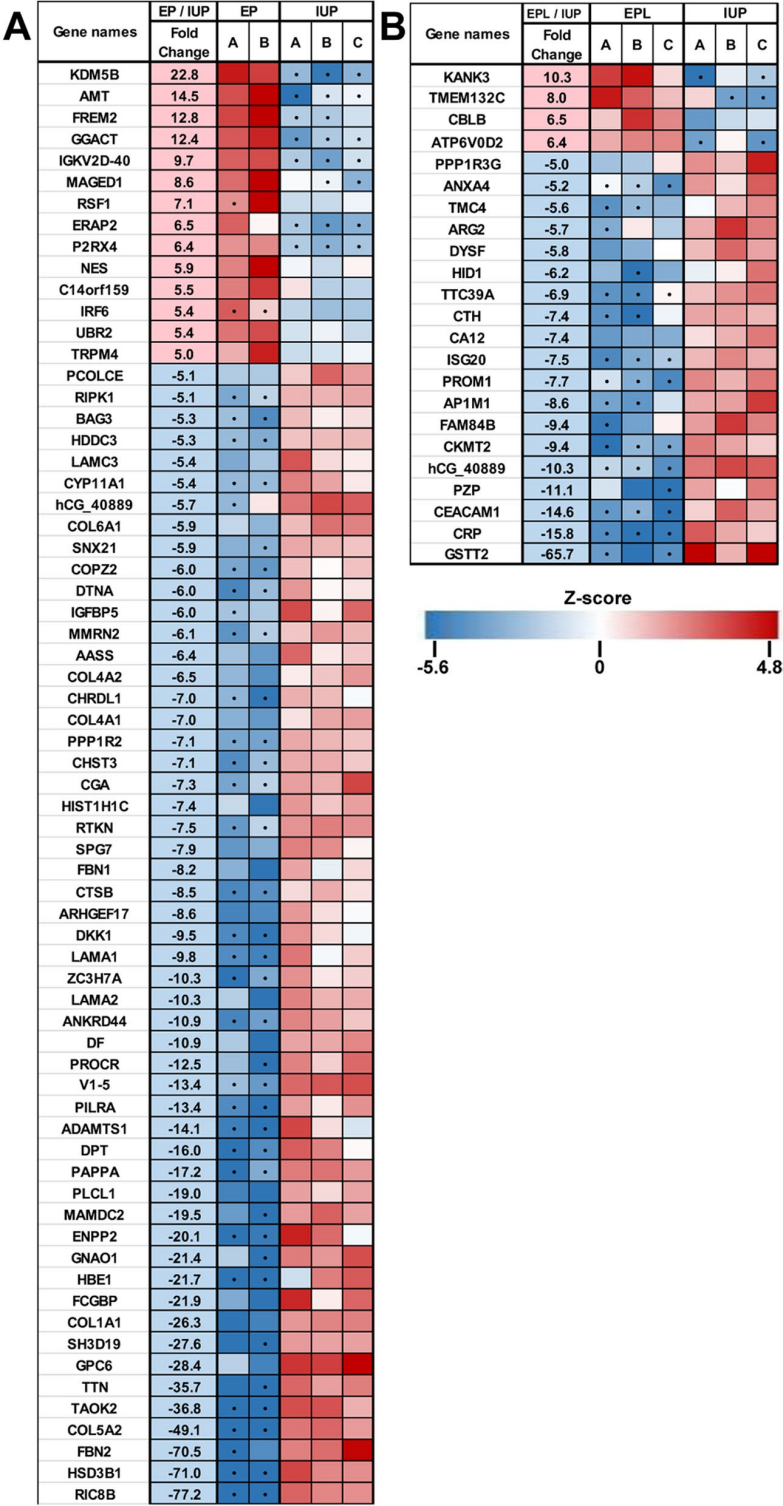
Heatmap of the largest significant changes in decidua tissues. Z-scores for relative protein quantities of proteins showing at least fivefold changes ( Student’s t-test p-value ≤ 0.05). Red = increased protein amount; blue = decreased protein amount; white = average intensity (no change); • = zero values that were replaced by imputed values for statistical analysis (see Materials and Methods). **a.** Comparison of EP and IUP decidua specimens. **b.** Comparison of EPL and IUP trophoblast specimens

**Fig. 4:**
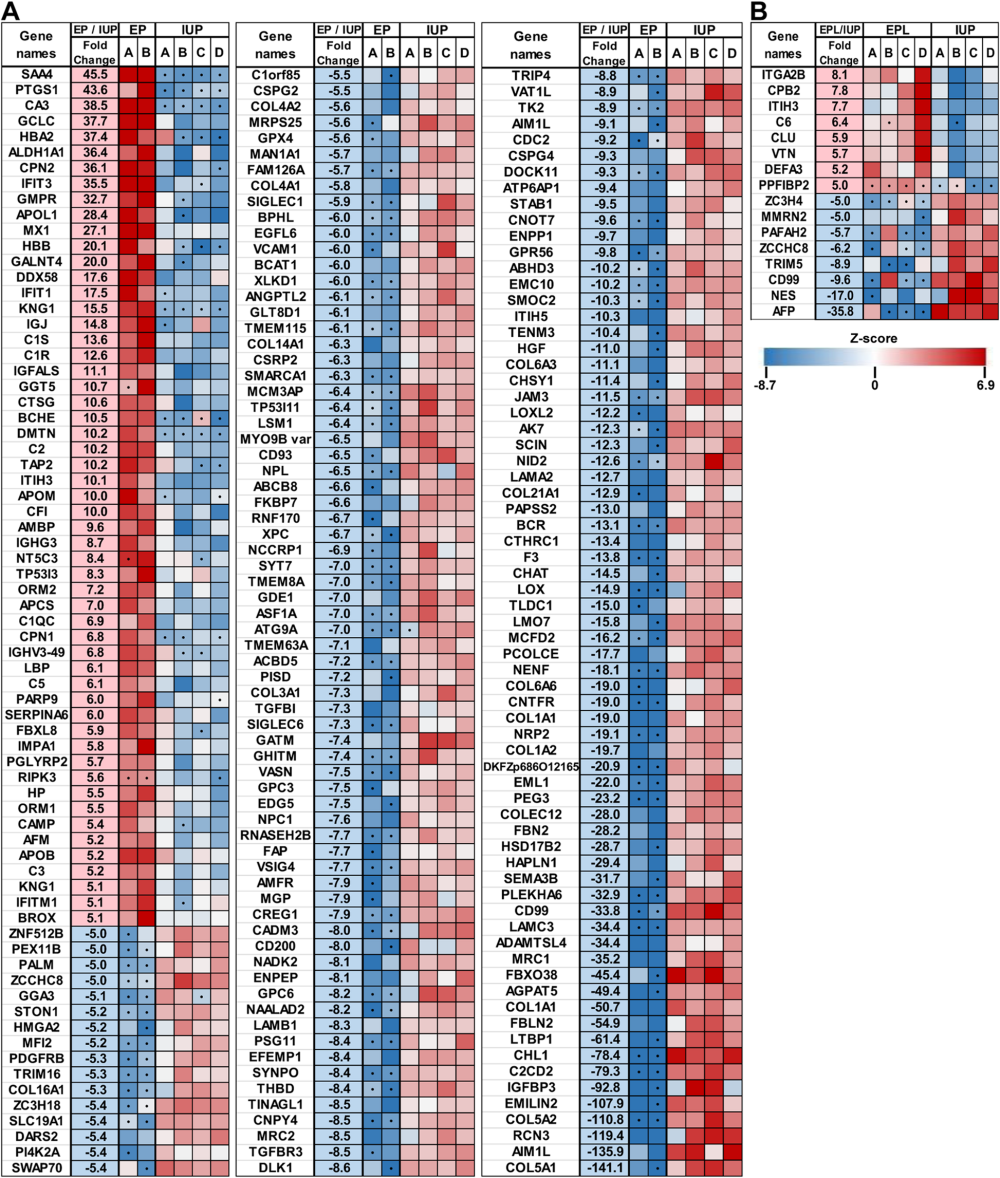
Heatmap of the largest significant changes in trophoblast tissues. Z-scores for relative protein quantities of proteins showing at least fivefold changes (Student’s t-test p-value ≤ 0.05). Red = increased protein amount; blue = decreased protein amount; white = average intensity (no change); • = zero values that were replaced by imputed values for statistical analysis (see Materials and Methods). **a.** Comparison of EP and IUP trophoblast specimens. **b.** Comparison of EPL and IUP trophoblast specimens

Within each tissue-type, pairwise comparisons of pregnancy location (EP vs. IUP) or viability of intrauterine pregnancy (EPL vs. IUP) were performed and protein changes of ≥ threefold and a Student’s t-test p-value ≤ 0.05 were considered significant. In the decidua, 125 protein quantities (2.6% of the proteome) were significantly different between EP and IUP, whereas EPL and IUP decidua were more similar with only 68 (1.4%) differences (Fig. 2a). A similar degree of protein changes was observed between EPL and IUP trophoblasts with 66 (1.4%) differences (Fig. 2a). The largest differences were observed for the EP and IUP trophoblasts with 344 (7.2%) differences (Fig. 2a). For both tissue types, there was minimal overlap (~ 2- 4%) between the changes observed when comparing location (EP/IUP) and intrauterine viability (EPL/IUP) (Fig. 2b and c). Heat maps of the top protein changes based on either location or viability illustrate a high level of consistency across individual specimens within each group for both decidua (Fig. 3) and trophoblast (Fig. 4) tissues.

### Determination of major cell types in the trophoblast and decidua proteomes

To determine the degree of similarities among cell types for the different pregnancy tissues, and whether major variations in cell types in any of the tissue specimens might have affected the proteome, we cross-referenced our tissue datasets with a scRNA-seq analysis of human first-trimester placental villi and decidual cells. The published placental villi data files contained average expression for nine cell types and 17,106 genes and the decidua dataset contained 13 cell types and 16,967 genes. Our proteome datasets were matched to the corresponding reference dataset using gene names which mapped 4,417 genes for trophoblasts and 4,440 genes for decidua. Pie charts representing the summed intensities of mapped proteins identified in each tissue proteome show very similar distributions of cell types among all pregnancy outcomes (Supplementary Fig. S3).

### Biological pathway analysis reveals specific KEGG pathway enrichment

**Fig. 5:**
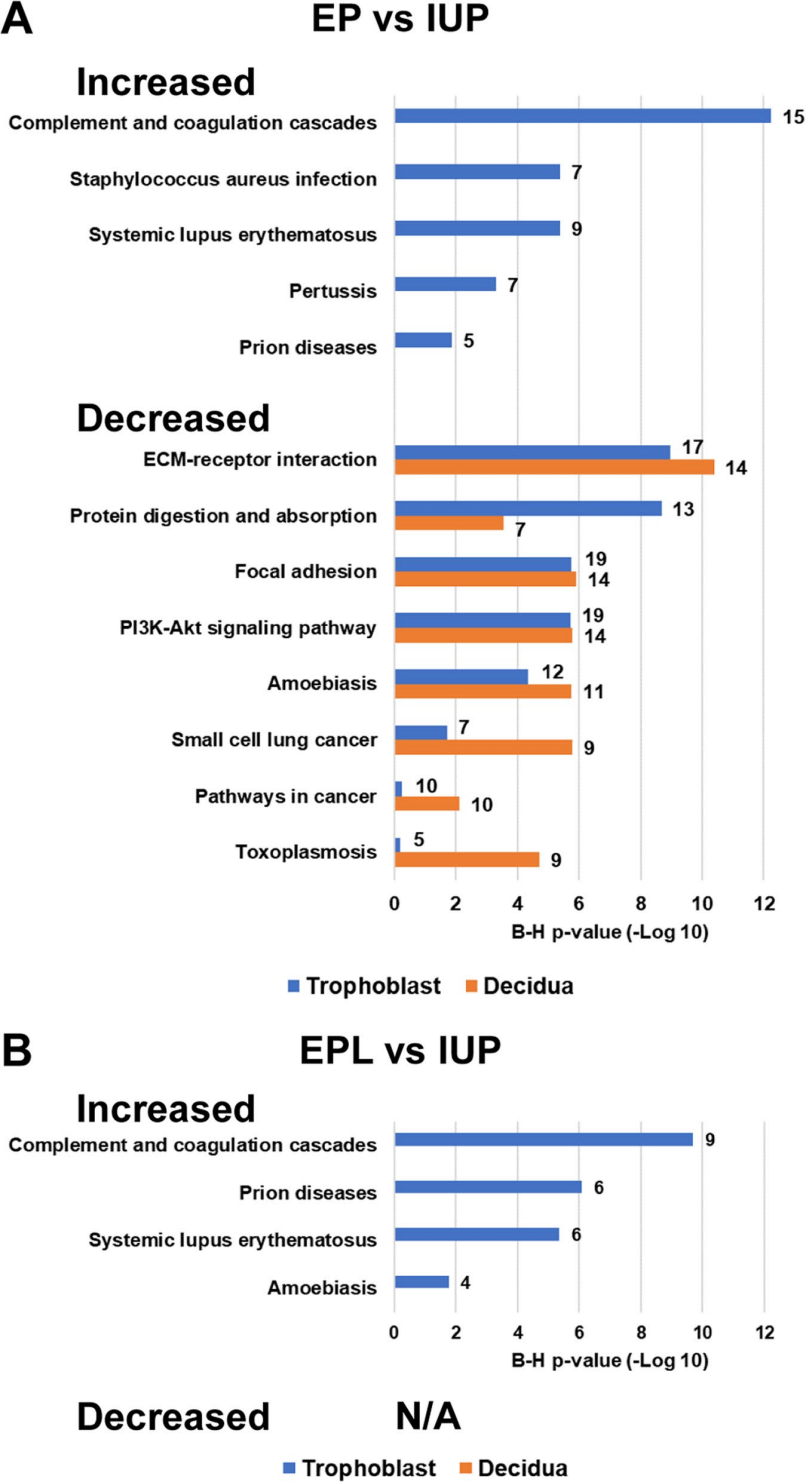
KEGG pathway enrichment analysis of significantly changed proteins. KEGG pathway enrichment was performed using DAVID. **a.** Enriched pathways identified when comparing EP vs. IUP trophoblast or decidua specimens **b.** Enriched pathways identified when comparing EPL vs. IUP trophoblast specimens. The numbers next to each bar represent the number of identified genes related to the corresponding pathway. N/A: Not Applicable; no significant enrichment was identified

When comparing protein groups increased in either EP vs. IUP or EPL vs. IUP trophoblasts, a DAVID analysis of KEGG pathway enrichment identified pathways involved in immune responses such as the complement and coagulation cascades, as well as more specific host-immune response pathways related to infection and disease (Fig. 5a and b). In this analysis the complement cascade was identified as the top increased cellular pathway in both EPL vs. IUP and EP vs. IUP comparisons, and notably enrichment of this as well as other increased pathways were specific to the trophoblast. There was no significant enrichment for protein groups decreased in EPL vs. IUP for either tissue-type (Fig. 5b). In contrast, for both trophoblast and decidua, proteins decreased in EP vs. IUP are enriched for pathways including extracellular matrix (ECM) receptor interactions, focal adhesion, the P13K-Akt signaling pathway and metabolic pathways such as protein digestion/absorption (Fig. 5a).

In decidua specimens, there was no significant pathway enrichment for proteins increased in EP or EPL compared to IUP. However, a number of proteins decreased in EP decidua relative to IUP were correlated to toxoplasmosis, and other proteins were involved in cancer-related pathways. (Fig. 5a).

### Protein–protein interaction networks reveals specific GO Biological processes for comparison of EP and IUP

**Fig. 6:**
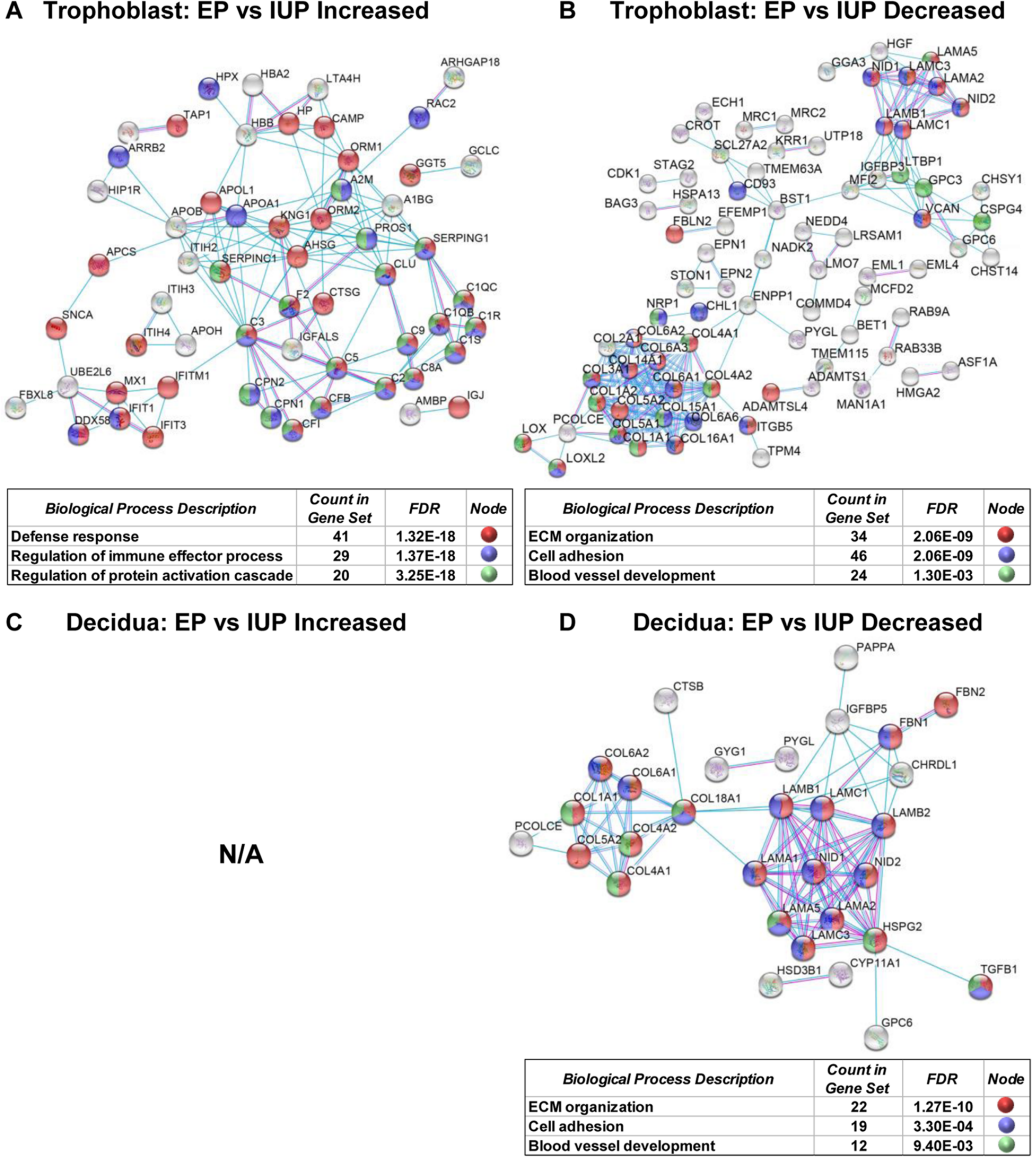
Protein–protein Interaction Networks for EP vs. IUP. Interaction networks derived from STRING with selected top Gene Ontology (GO) Biological Processes color coded as indicated in the tables below each network. **a.** Interactions identified when comparing increases in EP vs. IUP trophoblast specimens. **b.** Interactions identified when comparing decreases in EP vs. IUP trophoblast specimens. **c.** Interactions identified when comparing increases in EP vs. IUP decidua specimens. N/A: Not Applicable; no significant interactions were identified. **d.** Interactions identified when comparing decreases in EP vs. IUP decidua specimens

Protein–protein interaction networks and GO biological processes enrichments were also evaluated using STRING, which produced results that were generally consistent with the DAVID analyses summarized above, while providing a different graphical perspective of the data. Specifically, for trophoblast proteins increased in EP vs. IUP, there was enrichment in immune response and protein activation cascades (Fig. 6a). There were also significant decreases in EP specimens of both tissue-types for ECM remodeling, cell adhesion, and blood vessel development (Fig. 6b, d). However, these decreases are most dramatic in the EP trophoblast. Overall, no significant networks were identified when comparing EPL vs. IUP in either tissue, and in decidua, increases in EP vs. IUP also resulted in no significant interactions (Fig. 6c).

### Key hormones that regulate embryonic development are most extensively affected in EP vs. IUP trophoblasts

In an IPA Upstream Regulator Analysis, trophoblast EP vs. IUP comparisons predicted the largest number of upstream regulators with 45 molecules showing a predicted activation state and 45 showing an inhibited state (Supplementary Table 1). In comparison, the decidua EP vs. IUP analyses predicted 11 molecules showing an activation state and 15 molecules showing an inhibited state (Supplementary Table 2).

**Fig. 7:**
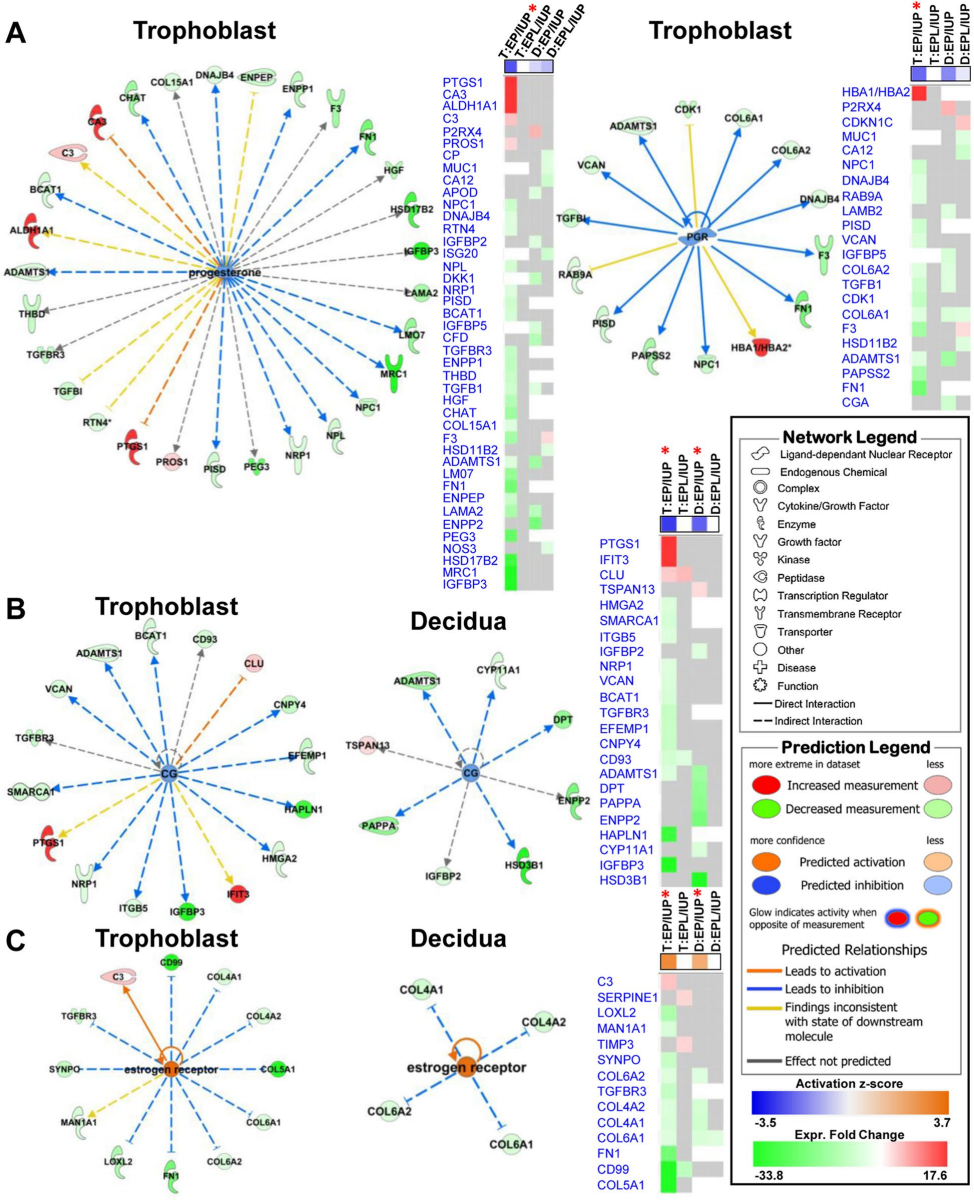
Significant interactions between predicted upstream hormone regulators and protein changes for EP vs. IUP. **a.** Left: network of progesterone regulated proteins in EP vs. IUP trophoblast specimens and heatmap showing related protein changes for pairwise comparisons in both tissues. Right: progesterone receptor (PGR) network and corresponding heatmap. **b.** Networks and heatmap for **c**horionic gonadotropin (CG) regulated proteins identified in the trophoblast and decidua. **c.** Networks and heatmap of estrogen receptor regulated proteins identified in the trophoblast and decidua. On heatmaps: red asterisks indicate activated (z-score ≥ 2; orange highlight in box) or inhibited (z-score ≤  − 2; blue highlight in box) upstream regulators. Increased and decreased protein changes are highlighted in red and green. T: trophoblast; D: decidua. Other network and prediction parameters are defined in the network legend

Several key upstream hormone regulators and receptors related to pregnancy and embryonic development are shown in Fig. 7. Progesterone and progesterone receptor (PGR) were both predicted to be significantly inhibited in EP vs. IUP trophoblast specimens. Figure 7a demonstrates a network of progesterone and its 30 downstream targets, as well as PGR and its 14 downstream targets in the EP vs. IUP trophoblast dataset. Corresponding heatmaps compare related protein changes of reported downstream targets across each pairwise comparison (Fig. 7a-c).

Chorionic gonadotropin (CG) was predicted to be significantly inhibited in both the trophoblast and decidua EP vs. IUP comparisons and the trophoblast had more downstream targets identified (Fig. 7b). Estrogen receptor activation prediction is presented in Fig. 7c.

### Other upstream regulators and their effects on tissue development

**Fig. 8:**
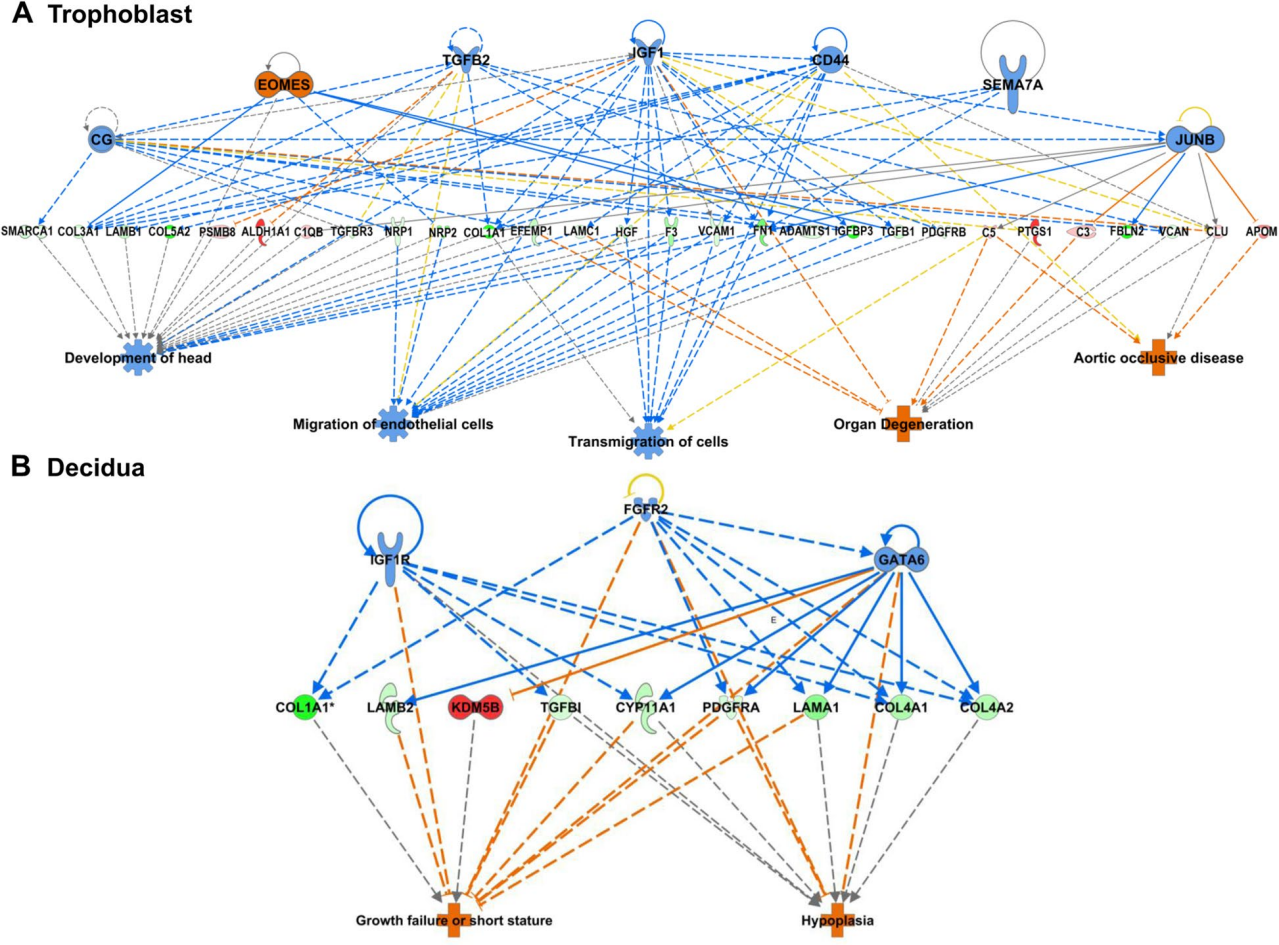
Top EP vs. IUP regulator effects networks from Ingenuity Pathway Analysis (IPA)**.** Upper network tier: predicted upstream regulators; middle tier: the genes identified in the proteome analysis that connect the upstream regulators and downstream related diseases and functions; bottom tier: related diseases or functions. **a.** Top regulator effects network identified in EP vs. IUP trophoblasts (Consistency score = 6.80). **b.** Top regulator effects network identified in EP vs. IUP decidua (Consistency score = 3.33). For other network and prediction legends, see Fig. 7

Further examination of significant EP vs. IUP upstream regulators in the trophoblast by the IPA Regulator Effects algorithm predicted a top network of regulators including CG, EOMES, TGFB2, IGF1, CD44, SEMA7A, and JUNB (consistency score 6.8; Fig. 8a), and 28 downstream targets which may regulate decreased development and cell migration, and increased organ degeneration and aortic disease. The top upstream regulators identified in the EP vs. IUP decidua analyses include IGF1R, FGFR2, and GATA6 (consistency score 3.3, Fig. 8b), which regulate nine downstream targets including several collagens and laminins, and have a predicted functional effect on growth failure and tissue hypoplasia.

## Discussion

This study used in-depth label-free proteomics to characterize and compare the maternal–fetal interface, consisting of the maternal decidua and the invading fetal trophoblast from different early pregnancy outcomes, including a comparison of nonviable EPL vs. viable IUP as well as EP vs. IUP. This allows comparison of the two different tissue proteomes for each clinical outcome (trophoblast and decidua) as well as comparisons of the proteomes of each tissue across different clinical outcomes.

Overall, the numbers of high confidence proteins identified in the trophoblast and decidua were similar with a high degree of overlap. The comparison of each tissue between pregnancy outcome resulted in substantial identifiable differences. In the decidua these differences were greater (2.6% of the proteome) between EP and IUP than between EPL and IUP (1.4%) suggesting high specificity of protein changes related to these different pregnancy conditions. However, the largest difference was noted in the trophoblast in women with an EP vs. that of an IUP. It is interesting that when comparing EPL and IUP, the embryo is in physical contact with the decidua tissue (modified uterine endometrium) and therefore similar proteomes are not surprising. In contrast, for EP, the embryo is in a disparate location; however, the decidua proteomes are still very similar, whereas the trophoblast proteomes are quite different. This suggests that the properties of the decidua are driven by systemic changes rather than proximity of the embryo, whereas local environment and tissue interactions much more profoundly affect the trophoblast. The similarity between nonviable and viable intrauterine trophoblasts (EPL vs. IUP) further suggests that local environment rather than health of the embryo is a primary driver of tissue composition.

Determination of major cell types in the trophoblast and decidua proteomes show very similar distributions of cell types among all pregnancy outcomes, indicating that there are not extensive changes in cell type compositions or differences in contamination of a particular cell type in surgical specimens that might otherwise bias the proteomes identified. The GO and KEGG pathway analyses revealed specific enrichment for EP vs. IUP trophoblasts in immune response pathways such as the complement cascade as well as more specific host-immune response pathways. It is well known that during pregnancy, highly regulated immune responses occur between the invading trophoblast and the receptive maternal decidua [8, 28, 29]. Immune regulation during early pregnancy is necessary to ensure successful implantation, and a responsive maternal immune system is necessary to protect the mother and the fetus against external pathogens and environmental injury [8]. In EP, alterations in immune cells and inflammatory responses in the fallopian tube are thought to be a major predisposing condition due to a higher level of pro-inflammatory markers which cause persistent tubal damage. As a result, inflammatory signals may guide the embryo toward the inflammatory site, and increased immune cells create an environment conducive to embryo implantation [30, 31]. Moreover, a balanced complement and coagulation system is recognized as being an essential immune response factor in normal placental and fetal development [28, 32, 33]. Dysregulation of the complement system has been implicated in adverse pregnancy outcomes such as recurrent miscarriage and pre-ecclampsia [33–36]; however, the role of the complement cascade in EP is less established.

For both trophoblast and decidua, proteins decreased in EP vs. IUP are involved in pathways including ECM-receptor interactions, focal adhesion, P13K-Akt cell cycle pathway, and protein digestion/absorption metabolic pathways. Several of these pathways are known to be related to pregnancy and trophoblast invasion. Specifically, PI3K-Akt signaling is associated with developmental processes such as cell growth, proliferation, and migration, while focal adhesion signaling also plays a crucial role in trophoblast migration and invasion [37]. Overall, the reduced cell-ECM interactions and reduced cell signaling for EP are consistent with the disparate locations of these normally interacting tissues in EP.

An additional pathway of potential interest enriched in proteins decreased in EP vs. IUP decidua was the toxoplasmosis pathway. This pathogenic pathway may be related to a host’s immune response to infection and disease. Additionally, other proteins decreased in EP compared to IUP decidua were involved in cancer-related pathways. These pathways were likely identified in this study because it is known that many proliferative, invasive, and immune tolerance mechanisms that support a pregnancy are also utilized by malignancies to establish a nutrient supply and evade or alter the host’s immune response [8, 38]. Therefore, it is reasonable to expect that a normal IUP may overlap with these pathway categories while in EP, when an “invading” embryo is in a distal location, these processes are deficient.

In an Upstream Regulator Analysis, we also noted that key hormones that may regulate embryonic development are affected in the trophoblast of EP and IUP. Progesterone and PGR were both predicted to be significantly inhibited in EP vs. IUP trophoblast specimens while both regulators were not identified as having a significant activation state in any of the decidua comparisons. A comparison heatmap of progesterone’s 30 downstream targets in each pairwise comparison shows that the related protein changes are significantly affected in the trophoblast EP vs. IUP comparison. Likewise, PGR has 14 downstream targets most affected in the trophoblast EP vs. IUP. PGR had a significant p-value of overlap (p ≤ 0.05) in the decidua EP vs. IUP analysis, but the predicted activation state was not significant.

CG was predicted to be significantly inhibited in both the trophoblast and decidua EP vs. IUP comparisons and the trophoblast had more downstream targets identified. CG regulates different protein targets in each tissue, with the only common target being ADAMTS1, a metalloproteinase responsible for ECM remodeling and known to play a role in maintaining decidualization, and also critical for embryo implantation in mice [39, 40]. Conversely, estrogen receptor was predicted to be activated in both the trophoblast and the decidua. However, it was more extensively affected in the trophoblast with 12 downstream targets compared to four in the decidua, and all targets in the decidua overlapped with those identified in the trophoblast (Fig. 7c). Evaluation of these pregnancy-related hormone regulators also shows a connection to ECM protein regulation. In the trophoblast, inhibition of progesterone and PGR predicts an inhibition of several collagens and fibronectin (Fig. 7a), while an activation of estrogen receptor also inhibits several collagens in both tissues (Fig. 7c).

The IPA Regulator Effects analyses of other upstream regulators of EP vs. IUP were consistent with the pregnancy-related hormone regulators described above. The 28 downstream targets in this network also include predicted inhibition of a number of ECM proteins and predicted activation of several immune response proteins. The overall functional effects identified in the trophoblast are a predicted inhibition in developmental processes and cell migration, and a predicted increase in organ or tissue degeneration and blood vessel disease. The predicted downstream effects in the decidua EP vs IUP comparison were an activation of growth failure and hypoplasia, or incomplete tissue development.

Limitations of our analysis were a small number of samples and contamination of two tissue samples with blood that severely limited depth of analysis (number of identified proteins) resulting in their exclusion from the data analysis. Additionally, EPL and IUP specimens were collected at the time of a clinical procedure; therefore, we were unable to specify if decidua specimens were from an area which has invasive trophoblast present (basalis) or from an area where there is no trophoblast invasion (pariatalis), or both. Due to the limited number of specimens available for analysis, this study should be considered exploratory, and therefore conservative statistical criteria were used. There was still some degree of blood contamination in the remaining trophoblast and decidua specimens which caused minor variability but did not dramatically affect depth of analysis and did not skew the results. We also acknowledge that the generic classifications generated by the IPA software do not necessarily precisely define biological changes specific for biological system being studied such as cancer-related pathways, but rather give an idea of the processes that are perturbed.

## Conclusions

This exploratory proteomics study used in-depth proteomics analysis to characterize the maternal–fetal interface from different early pregnancy outcomes, including a comparison of nonviable EPL vs. viable intrauterine pregnancy (IUP) and ectopic pregnancy (EP) vs. IUP. There were very few differences between a nonviable vs. viable intrauterine pregnancy for both tissue types. In contrast, large differences were observed between trophoblasts from EP and IUP, which is not surprising considering the substantially different locations of the embryos in these conditions. Pathway analyses showed that in both tissue types, proteins associated with extracellular matrix remodeling, cell adhesion and metabolic pathways were decreased in EP specimens compared with IUP. In trophoblasts, EP and EPL specimens both showed elevation of inflammatory and immune response pathways. Taken together, these results suggest that biomarkers and mechanisms of trophoblast function may be the best predictors of early pregnancy location and viability.

## Supplementary Information

**Additional file 1:** **Supplemental Table 1.** Predicted upstream regulators identified in the trophoblast.

**Additional file 2:** **Supplementary Table S2.** Predicted upstream regulators identified in the decidua.

**Additional file 3:** **Supplementary Fig. S1. **Overlap of trophoblast proteomes across specimens within each pregnancy outcome and between pregnancy outcomes. a. Overlap of protein identifications in IUP trophoblast tissue proteomes. b. Overlap of protein identifications in EPL trophoblast tissue proteomes. c. Overlap of protein identifications in EP trophoblast tissue proteomes. d. Overlap of protein identifications among all three outcomes for trophoblast tissue proteomes. IUP: intrauterine pregnancy; EPL: early pregnancy loss; EP: ectopic pregnancy.

**Additional file 4: Supplementary Fig. S2.** Overlap of decidua proteomes across specimens within each pregnancy outcome and between pregnancy outcomes. a.Overlap of protein identifications in IUP decidua tissue proteomes. b. Overlap of protein identifications in EPL decidua tissue proteomes. c. Overlap of protein identifications in EP decidua tissue proteomes. d. Overlap of protein identifications among all three outcomes for decidua tissue proteomes.

**Additional file 5: Supplementary Fig. S3.** Major cell types in trophoblast and decidua specimens. Trophoblast and decidua datasets were cross referenced with an scRNA-seq analysis of human first-trimester placental villi and decidual cells [27]. Pie charts and percentages represent summed intensities of proteins identified in the EP, IUP, and EPL tissue proteomes mapped by gene name to cell types from the reference dataset. a.Trophoblast cell types: villous cytotrophoblasts** (**VCT); syncytiotrophoblasts (SCT) ; extravillous trophoblasts (EVT) ; fibroblasts (FB) ; vascular endothelial cells (VEC) ; erythroblasts (EB) ; fetal macrophages (Hofbauer cells, HC). TPM Zero: proteins mapped to genes with zero expression in the scRNA analysis; Not Mapped: proteins not mapped to any genes in the scRNA analysis. b. Decidua cell types: decidualized stromal cells (DSC) ; smooth muscle cells (SMC) ; natural killer cells (NK) ; T cells (TC) ; antigen-presenting cells (APC) ; endometrial epithelial cells (EEC) ; lymphatic endothelial cells (LEC); vascular endothelial cells (VEC), TPM Zero: proteins mapped to genes with zero expression in the scRNA analysis; Not Mapped: proteins not mapped to any genes in the scRNA analysis.

## Data Availability

The mass spectrometry proteomics data have been deposited to the MassIVE data repository with the accession number MSV000088572.

## Abbreviations

IUP: Intrauterine pregnancy
EPL: Early pregnancy loss
EP: Ectopic pregnancy
LC–MS/MS: Liquid chromatography-tandem mass spectrometry
hCG: Human chorionic gonadotropin
GA: Gestational age
UPLC: Ultrahigh pressure liquid chromatography
RP-HPLC: Reverse phase-high pressure liquid chromatography
FDR: False discovery rate
LFQ: label-free quantitation
DAVID: The Database for Annotation, Visualization, and Integrated Discovery
IPA: Ingenuity Pathway Analysis
KEGG: Kyoto Encyclopedia of Genes and Genomes
GO: Gene Ontology
scRNA-seq: Single-cell ribonucleic acid sequencing
ECM: Extracellular matrix
PGR: Progesterone receptor
CG: Chorionic gonadotropin
