# Supplementary figures and images for: Proteome-defined changes in cellular pathways for decidua and trophoblast tissues associated with location and viability of early-stage pregnancy
Source: Reprod Biol Endocrinol. 2022 Feb 21;20:36. doi: 10.1186/s12958-022-00908-3 (PMC8862331; doi:10.1186/s12958-022-00908-3)

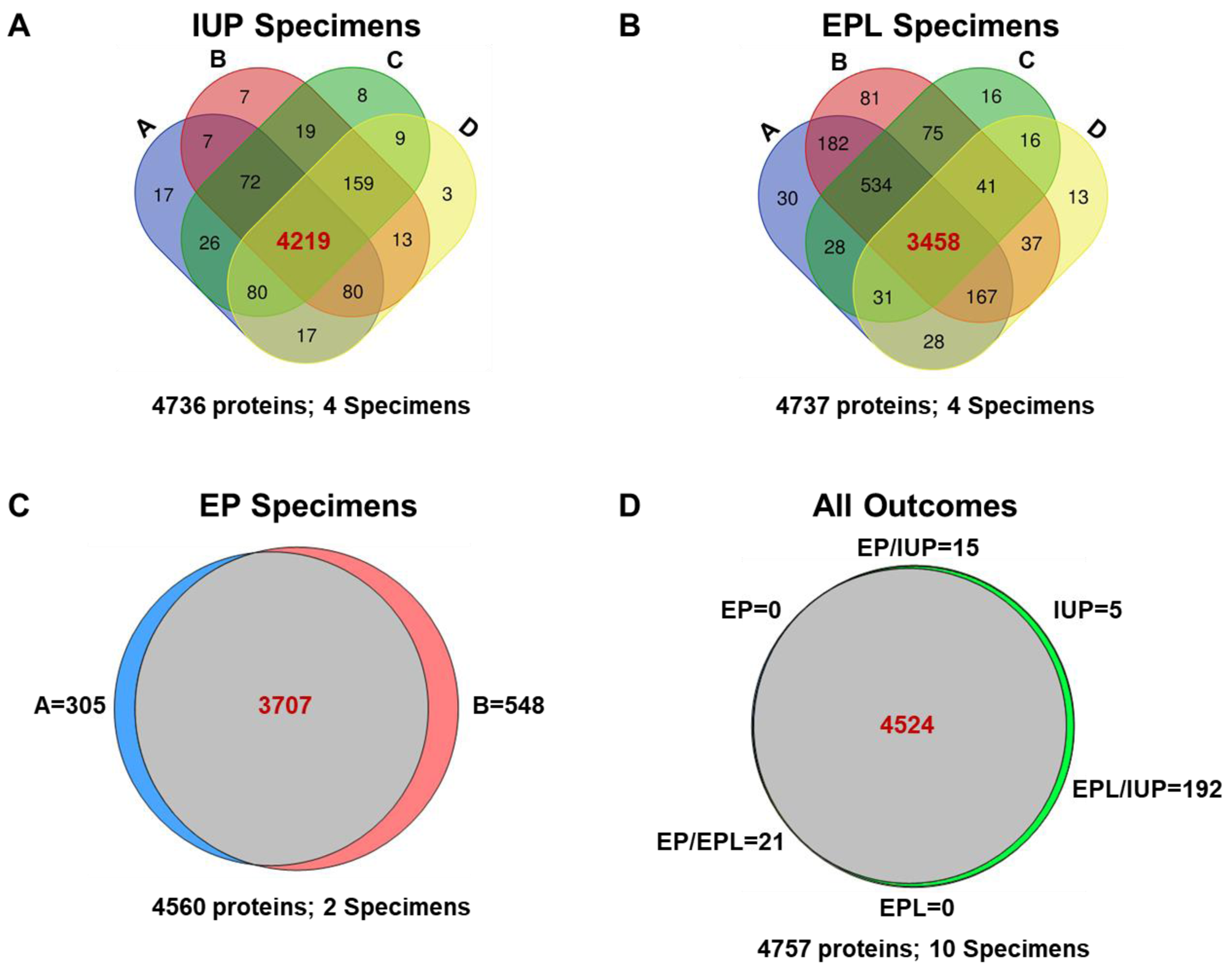

Supplement: Supplementary file 3 — Additional file 3: Supplementary Fig. S1. Overlap of trophoblast proteomes across specimens within each pregnancy outcome and between pregnancy outcomes. a. Overlap of protein identifications in IUP trophoblast tissue proteomes. b. Overlap of protein identifications in EPL trophoblast tissue proteomes. c. Overlap of protein identifications in EP trophoblast tissue proteomes. d. Overlap of protein identifications among all three outcomes for trophoblast tissue proteomes. IUP: intrauterine pregnancy; EPL: early pregnancy loss; EP: ectopic pregnancy. [file 12958_2022_908_MOESM3_ESM.tif]

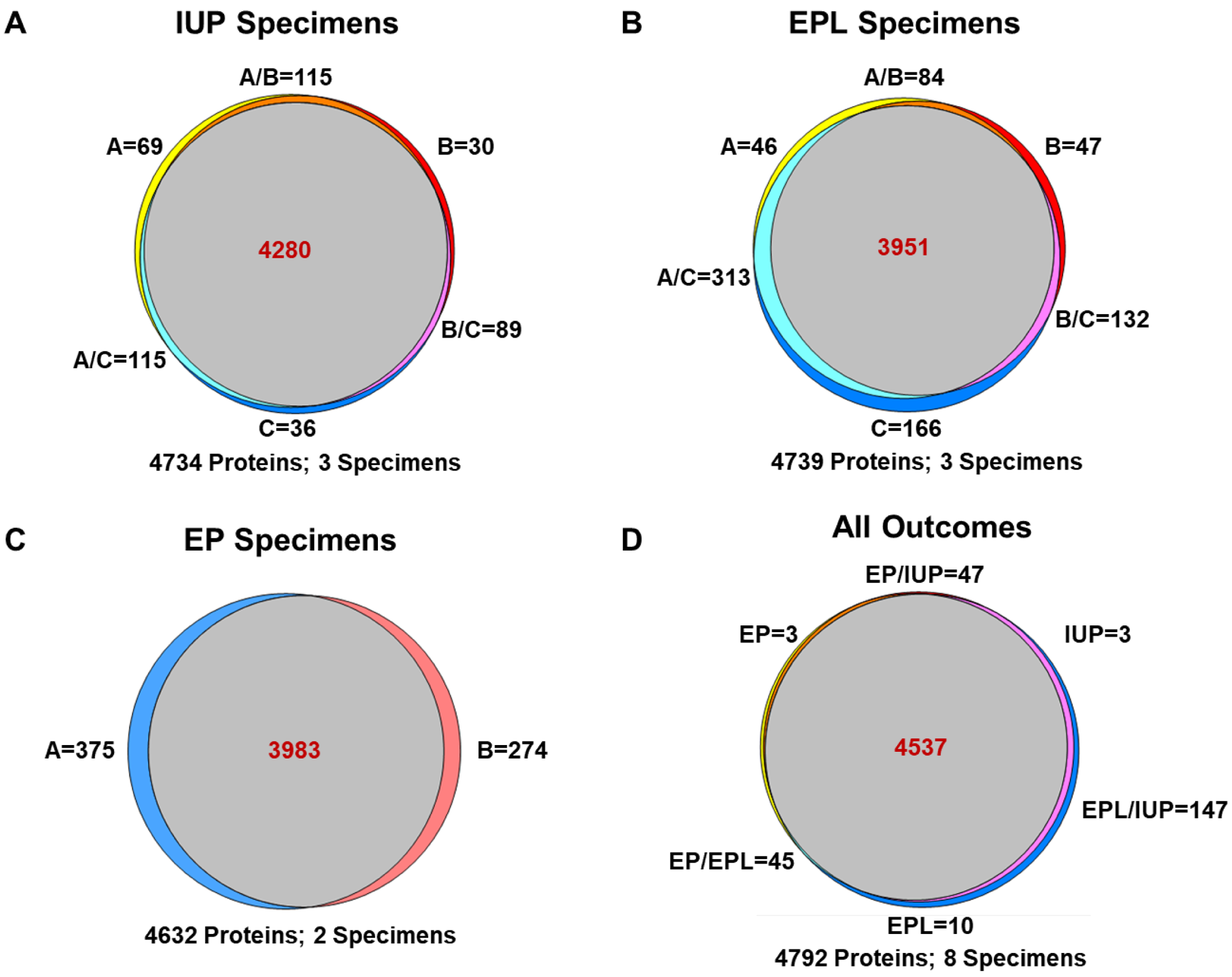

Supplement: Supplementary file 4 — Additional file 4: Supplementary Fig. S2. Overlap of decidua proteomes across specimens within each pregnancy outcome and between pregnancy outcomes. a.Overlap of protein identifications in IUP decidua tissue proteomes. b. Overlap of protein identifications in EPL decidua tissue proteomes. c. Overlap of protein identifications in EP decidua tissue proteomes. d. Overlap of protein identifications among all three outcomes for decidua tissue proteomes. [file 12958_2022_908_MOESM4_ESM.tif]

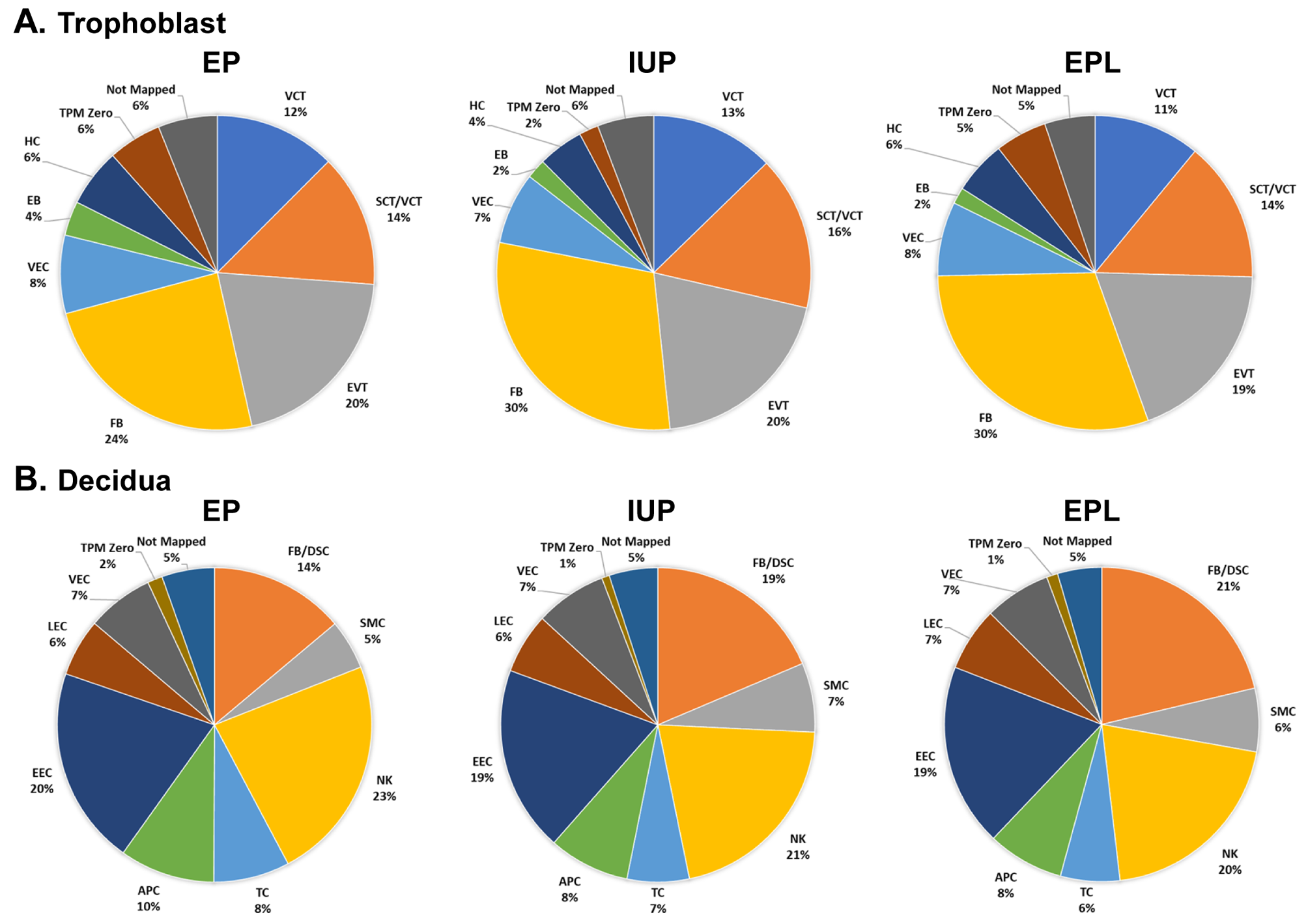

Supplement: Supplementary file 5 — Additional file 5: Supplementary Fig. S3. Major cell types in trophoblast and decidua specimens. Trophoblast and decidua datasets were cross referenced with an scRNA-seq analysis of human first-trimester placental villi and decidual cells [27]. Pie charts and percentages represent summed intensities of proteins identified in the EP, IUP, and EPL tissue proteomes mapped by gene name to cell types from the reference dataset. a.Trophoblast cell types: villous cytotrophoblasts (VCT); syncytiotrophoblasts (SCT) ; extravillous trophoblasts (EVT) ; fibroblasts (FB) ; vascular endothelial cells (VEC) ; erythroblasts (EB) ; fetal macrophages (Hofbauer cells, HC). TPM Zero: proteins mapped to genes with zero expression in the scRNA analysis; Not Mapped: proteins not mapped to any genes in the scRNA analysis. b. Decidua cell types: decidualized stromal cells (DSC) ; smooth muscle cells (SMC) ; natural killer cells (NK) ; T cells (TC) ; antigen-presenting cells (APC) ; endometrial epithelial cells (EEC) ; lymphatic endothelial cells (LEC); vascular endothelial cells (VEC), TPM Zero: proteins mapped to genes with zero expression in the scRNA analysis; Not Mapped: proteins not mapped to any genes in the scRNA analysis. [file 12958_2022_908_MOESM5_ESM.tif]
